# Editorial: Perspectives on the ultrastructure and cell biology of parasitic protists

**DOI:** 10.3389/fcimb.2023.1293959

**Published:** 2023-09-22

**Authors:** Victor Midlej, Antonio Pereira-Neves

**Affiliations:** ^1^ Laboratório de Biologia Estrutural, Instituto Oswaldo Cruz/Fiocruz, Rio de Janeiro, Brazil; ^2^ Departamento de Microbiologia, Instituto Aggeu Magalhães, Fiocruz, Recife, Pernambuco, Brazil

**Keywords:** microscopy, toxoplasmosis, Chagas disease, malaria, cell biology, protozoa

## Introduction

Parasitic diseases are commonly found in various locations across the globe. Some of them do not encounter geographic barriers, having among their infected individuals living in developed and developing countries. The causative agents of these diseases with the greatest medical and social impact are protists, unicellular organisms that are well known for their peculiar structural organization, a consequence of an early divergence from the main lineages of eukaryotic evolution. Many of these unusual features are adaptative mechanisms for a parasitic lifestyle and serve as potential pharmacological targets.

In this editorial, we briefly introduce a small collection of articles that bring contributions to different aspects of the Cell Biology of protozoa: (1) raising the state of the art in endoplasmic reticulum biology in trypanosomatids; (2) presenting new molecular components in *Toxoplasma gondii* and two new potential anti-*T. gondii* drugs*;* and (3) revealing new insights into the ultrastructure of the sexual stage of *Plasmodium falciparum*.

## Perspectives of ultrastructure and cell biology in trypanosomatids

The endoplasmic reticulum (ER) is a crucial organelle in eukaryotic cells and its importance cannot be overstated, as it participates in functions ranging from protein processing to responses to cellular stress. The role of this organelle in cell death pathways linked due to the reticulum connection with protein quality control and cellular homeostasis has been demonstrated. Sandes et al. reviewed the basic aspects of ER biology, organization, and quality control in trypanosomatids. This review brings to light important aspects about the cell biology of trypanosomatids in relation to the organization of the ER in *Trypanosoma cruzi*, *Trypanosoma brucei* and *Leishmania* sp. Different classifications of this organelle are evident with regard to: (i) the traditional role linked to the processing of proteins, lipids and carbohydrates, in addition to ion storage; (ii) place of exchange, referring to contact sites; (iii) endosymbiont aspects, when analyzing the regions where exogenous organisms settle. Furthermore, this work discusses the role of ERAD (Endoplasmic Reticulum-Associated Degradation) as a multi-step quality control mechanism that requires a sophisticated set of components to remove aberrant proteins that threaten the ER and, therefore, cellular balance.

## Perspectives of ultrastructure and cell biology in Apicomplexa

The Apicomplexa phylum comprises parasitic single-celled organisms known for their complex life cycles and specialized features, including the unique apical complex for host cell invasion and the apicoplast organelle, often targeted for drug development. They encompass significant human and veterinary pathogens like *Plasmodium* and *Toxoplasma*, with complex, multi-host life cycles and varying degrees of host specificity, sometimes developing drug resistance. Extensive research efforts focus on understanding their biology and developing interventions for the diseases they cause.

In contrast to apical complex, the basal complex (BC) is much less prominent, and the studies on its functions and organization are scant. BC is composed of juxtaposed rings located beneath the basal end of the parasite’s internal membrane complex that extends beneath the plasma membrane along the cell body (Hu, 2008). The identification of the molecular framework that composes this complex is extremely important for understanding its role in the parasite’s biology and as future targets in chemotherapy.

In this Research Topic, Roumégous et al. very elegantly showed the localization e function of 9 BC components from a total of 114 proteins, using elaborate techniques such as expansion microscopy, FRAP and electron microscopy, indicating new subdomains of this complex. Although none of these components proved to be essential for the growth of the parasite, some of them participate in the constriction of the BC, in gliding motility, in the formation and maintenance of the intravacuolar parasite connection.

*Toxoplasma gondii* is one of the best-known Apicomplexa organisms. Responsible for causing toxoplasmosis, it is currently still the target of searches for effective chemotherapeutic agents, as the disease has no cure (Martins-Duarte et al., 2021). Available drugs may not eliminate the parasite, leading to prolonged treatment and potential side effects. Drug resistance in some strains of the parasite further complicates therapy. The immune status of the host and the potential harm to a developing fetus during pregnancy must be considered, making treatment decisions complex. A study bringing together 152 small molecules obtained from the National Drug Screening Center in China associated with the autophagy death pathway revealed 38 compounds with significant *in vitro* activity against *T. gondii*. Among these, Hua et al. showed that two compounds, CGI-1746 and JH-II-127, exhibited a growth inhibitor effect in tachyzoites without affect the viability of the host cells. They demonstrated that CGI-1746 inhibited the invasion, egress and especially the gliding capacity of the parasites, while JH-II-127 seriously damaged the morphology of mitochondria that may be related to the damage to the mitochondrial electron transport chain.

*Plasmodium falciparum* is the human’s deadliest parasitic protist, responsible for the most severe form of malaria worldwide, especially in sub-Saharan Africa. The clinical manifestations only occur while the parasite is undergoing asexual reproduction within the humans’ erythrocytes. A small proportion of these asexual stages enters the sexual pathway and develops into male and female sexual forms called gametocytes which are essential for transmission of *P. falciparum* from humans to *Anopheles* mosquito vector. To survive inside erythrocytes, the parasite needs to remodel the host cell architecture; one of these dramatic changes is the formation of multiple and complex membranous compartments and networks called exomembrane structures (EMS) (Sherling and van Ooij, 2016). EMS are important components of *Plasmodium* biology, acting as key players involved in the sorting and trafficking of parasite proteins into the host cell cytosol (Mundwiler-Pachlatko and Beck, 2013). Maurer’s clefts (MC) are the best characterized part of the EMS, consisting of membrane-limiting structures scattered throughout the cytoplasm of *P. falciparum* infected erythrocytes. EMS are better studied in asexual stages of the parasite, and data concerning their origin and ultrastructural organization in gametocytes are scant and ongoing debate. In that sense, Hayakawa et al. provide relevant information for this field of study in an interesting work. Using different and complementary imaging techniques, Hayakawa et al. revealed a wide variety of complex membranous structures induced by *P. falciparum* gametocytes and compared these structures with those present in erythrocytes infected with asexual stages of the parasite. Their multi-imaging methods were also successful in revealing a beautiful 3D architecture of these intricate EMS inside gametocytes. The authors took the advantage of an elegant unroofing technique, previously established by themselves to study MC in asexual stages of *Plasmodium* using electron microscopy, to obtain stereoscopic data of EMS in gametocyte-infected erythrocytes, providing unique ultrastructural data and knowledge for upcoming cell biological studies. Hayakawa et al. highlighted that the classical MCs found in parasites’ asexual life stages are absent in the gametocytes; however, “balloon/pouch”-like structures budding from the parasitophorous vacuole membrane, some of which contained more layers of other “balloons”, are seen in gametocytes. Finally, the authors discuss that *P. falciparum* may remodels EMS in erythrocytes as an adaptive strategy for stage-specific biological activities during its sexual development.

## Closing remarks

We expect that the papers presented in this Research Topic highlights the importance of conceiving parasitic protists, in addition to their medical and veterinary seriousness, as interesting cell biology models. The understanding of the parasite cell biology and its interactions with the hosts is imperative to generate new and more effective diagnostic methods and treatment for those diseases. We also hope that the works highlighted herein reinforce the role of imaging and ultrastructural techniques as a valuable tool in understanding the parasite’s biology.

## Author contributions

VM: Writing – original draft, Writing – review & editing. AP: Writing – original draft, Writing – review & editing.
